# Supplementary figures and images for: Long-read sequencing to interrogate strain-level variation among adherent-invasive Escherichia coli isolated from human intestinal tissue
Source: PLoS One. 2021 Oct 28;16(10):e0259141. doi: 10.1371/journal.pone.0259141 (PMC8553045; doi:10.1371/journal.pone.0259141)

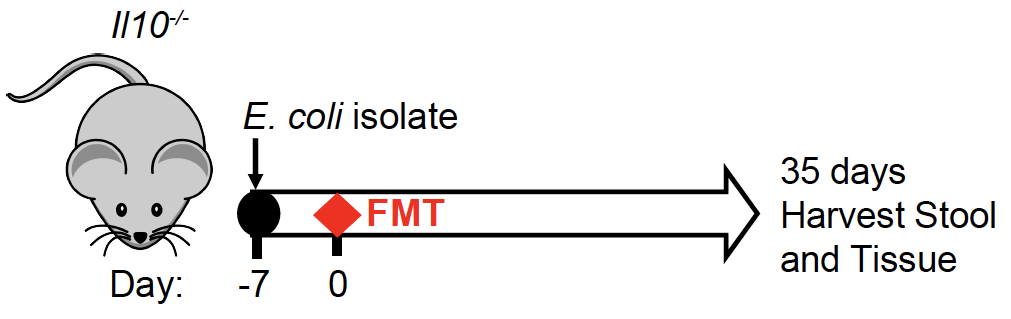


S1 Figure. Timeline for mouse experiment to determine colonize of individual *E. coli* isolates.

Supplement: S1 Fig — (DOCX) [file pone.0259141.s001.docx]
