# Supplementary material for: Long-read sequencing to interrogate strain-level variation among adherent-invasive Escherichia coli isolated from human intestinal tissue
Source: PLoS One. 2021 Oct 28;16(10):e0259141. doi: 10.1371/journal.pone.0259141 (PMC8553045; doi:10.1371/journal.pone.0259141)
Supplement: S2 Fig — A. Timeline for mouse experiment for colonization of CU42ET-1/D5 or HM670/C2. B. Levels of E. coli in stool over time (n = 7 in CU42ET-1/D5 and n = 8 in HM670/C2). Data point indicates the mean and error bars indicates the standard deviation (**p<0.01 Mann-Whitney test). If not otherwise noted, there is no significance. (DOCX) [file pone.0259141.s002.docx]

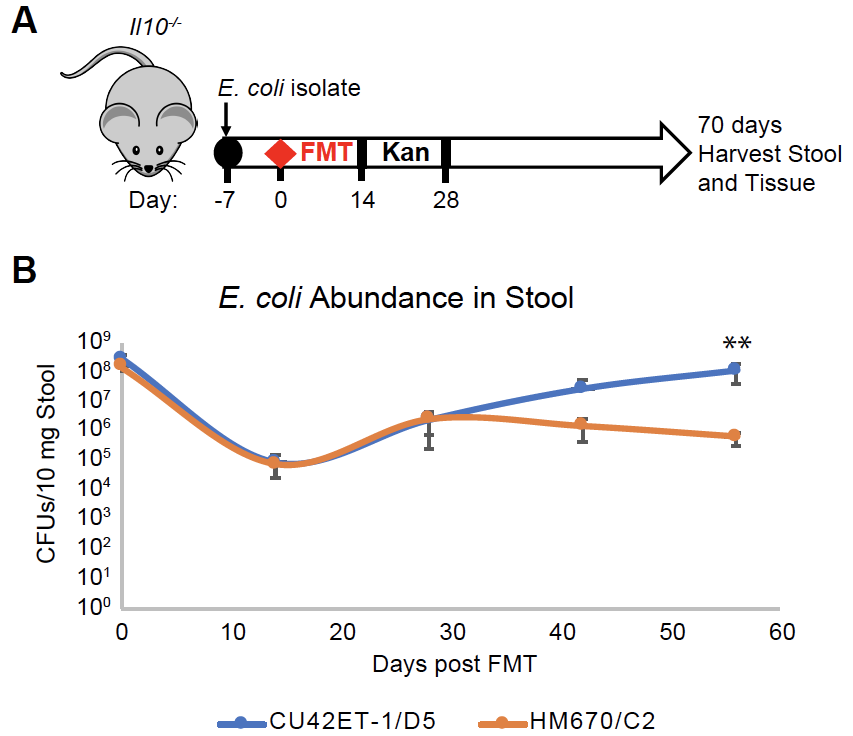


S2 Figure. A. Timeline for mouse experiment for colonization of CU42ET-1/D5 or HM670/C2. B. Levels of *E. coli* in stool over time (n=7 in CU42ET-1/D5 and n=8 in HM670/C2). Data point indicates the mean and error bars indicates the standard deviation (**p<0.01 Mann-Whitney test). If not otherwise noted, there is no significance.
