# Supplementary material for: Long-read sequencing to interrogate strain-level variation among adherent-invasive Escherichia coli isolated from human intestinal tissue
Source: PLoS One. 2021 Oct 28;16(10):e0259141. doi: 10.1371/journal.pone.0259141 (PMC8553045; doi:10.1371/journal.pone.0259141)
Supplement: S1 Table — Values indicate CFUs/10 mg of tissue. (DOCX) [file pone.0259141.s003.docx]

| **Strain** | **Mouse Gender** | **Ileal Tissue** | **Cecal Content** | **Colon Content** | **Mucus Layer** | **Colon Tissue** |
| --- | --- | --- | --- | --- | --- | --- |
| CU39ES-1/A1 | M | 65957 | 3916667 | 8588235 | 3556 | 12889 |
| CU39ES-1/A1 | F | 611 | 5882353 | 34166667 | 6500 | 10833 |
| CU532-9/A3 | M | 0 | 5806452 | 7058824 | 6667 | 455556 |
| LF82/B6 | M | 1875 | 232609 | 257732 | 200 | 1800 |
| LF82/B6 | F | 500 | 111111 | 74684 | 417 | 417 |
| CU568-3/C5 | M | 1250 | 40909091 | 5306122 | 24545 | 681818 |
| CU568-3/C5 | F | 0 | 0 | 71 | 0 | 0 |
| CU37RT-2/D2 | M | 0 | 0 | 0 | 0 | 0 |
| CU42ET-1/D5 | F | 28947 | 167857 | 320513 | 200 | 5133 |
| CU42ET-1/D5 | M | 107857 | 1333333 | ND | 1500 | 32308 |
| HM670/C2 | F | 0 | 34 | 0 | 0 | 188 |
| HM670/C2 | M | 13810 | 460317 | 422018 | 6583 | 25000 |
| NC101 | M | 500 | 39333 | 24615 | 0 | 70000 |

*Mice were all singly-housed.

* CU532-9/A3 F (dead), CU37RT-2/D2 F (dead), NC101 F (dead)

S1 Table. *E. coli* isolates colonize the lower gastrointestinal tract of *Il10^-/-^* mice with FMT competition. Values indicate CFUs/10 mg of tissue.
