# Supplementary material for: Long-read sequencing to interrogate strain-level variation among adherent-invasive Escherichia coli isolated from human intestinal tissue
Source: PLoS One. 2021 Oct 28;16(10):e0259141. doi: 10.1371/journal.pone.0259141 (PMC8553045; doi:10.1371/journal.pone.0259141)
Supplement: S2 Table — (DOCX) [file pone.0259141.s004.docx]

| **Mouse** | **Cage** | **Colon Content** | **Mucus Layer** | **Colon Tissue** |
| --- | --- | --- | --- | --- |
| CU42ET-1/D5 1 | 1 | 19,083,969 | 400,000 | 1,181,818 |
| CU42ET-1/D5 2 | 1 | 7,565,217 | 177,778 | 722,222 |
| CU42ET-1/D5 3 | 1 | 3,802,817 | 5,455 | 150,000 |
| CU42ET-1/D5 4 | 2 | 49,494,949 | 210,526 | 894,737 |
| CU42ET-1/D5 5 | 2 | 37,419,355 | 67,308 | 730,769 |
| CU42ET-1/D5 6 | 2 | 33,750,000 | 225,000 | 616,667 |
| CU42ET-1/D5 7 | 2 | 95,890,411 | 45,000 | 4,000,000 |
| HM670/C2 1 | 3 | 553,191 | 333 | 66,667 |
| HM670/C2 2 | 3 | 1,193,277 | 7,250 | 300,000 |
| HM670/C2 3 | 3 | 2,019,231 | 0 | 17,000 |
| HM670/C2 4 | 3 | 2,179,487 | 1,125 | 13,125 |
| HM670/C2 5 | 4 | 204,301 | 1,000 | 10,778 |
| HM670/C2 6 | 4 | 0 | 0 | 0 |
| HM670/C2 7 | 4 | 139 | 0 | 0 |
| HM670/C2 8 | 4 | 0 | 0 | 0 |

S2 Table. Consistent *E. coli* colonizer (CU42ET-1/D5) and inconsistent *E. coli* colonizer (HM670/C2) colonize the lower gastrointestinal tract of *Il10^-/-^* mice with FMT competition.
